# The Effect of Local Renin Angiotensin System in the Common Types of Cancer

**DOI:** 10.3389/fendo.2021.736361

**Published:** 2021-09-03

**Authors:** Moudhi Almutlaq, Abir Abdullah Alamro, Hassan S. Alamri, Amani Ahmed Alghamdi, Tlili Barhoumi

**Affiliations:** ^1^King Abdullah International Medical Research Centre, King Abdulaziz Medical City, Ministry of National Guard Health Affairs, Riyadh, Saudi Arabia; ^2^Biochemistry Department, College of Science, King Saud University, Riyadh, Saudi Arabia; ^3^Medical Research Core Facility and Platforms, King Saud bin Abdulaziz University for Health Sciences, Riyadh, Saudi Arabia

**Keywords:** renin angiotensin system, cancer, AT1R blockers, ACE inhibitors, Ang II, Ang 1-7, Urotensin II

## Abstract

The Renin Angiotensin System (RAS) is a hormonal system that is responsible for blood pressure hemostasis and electrolyte balance. It is implicated in cancer hallmarks because it is expressed locally in almost all of the body’s tissues. In this review, current knowledge on the effect of local RAS in the common types of cancer such as breast, lung, liver, prostate and skin cancer is summarised. The mechanisms by which RAS components could increase or decrease cancer activity are also discussed. In addition to the former, this review explores how the administration of AT1R blockers and ACE inhibitors drugs intervene with cancer therapy and contribute to the outcomes of cancer.

## Introduction

The Renin Angiotensin System (RAS), a hormonal system responsible for blood pressure hemostasis and electrolyte balance, chiefly consists of renin, angiotensinogen (AGT), angiotensin I (Ang I), angiotensin converting enzyme (ACE), angiotensin II (Ang II), angiotensin II type 1 receptor (AT1R) and angiotensin II type 2 receptor (AT2R), frequently referred to as the classical view of RAS. The alternative mainly consists of angiotensin 1-7 (Ang 1-7), MAS Receptor (MASR) and angiotensin converting enzyme 2 (ACE-2) (1). At low blood pressure, the kidneys release pro-renin into the blood, where it then converts to the active form – renin – which converts AGT to Ang I. Following this, Ang I is either converted to Ang II or Ang 1-7, by the actions of ACE and neprilysin, respectively. Furthermore, ACE2 is able to convert Ang II to Ang 1-7 (2–4). As an active hormone of RAS, Ang II increases blood pressure to a normal level. It does, however, have reverse effects in two types of G-protein couple receptors: AT1R and AT2R. The former is more common in adult tissues such as liver, brain, and kidney tissue, while the latter is predominantly present in foetal tissues, the ovary, and uterus (2).

In those suffering with hypertension, the overactivation of RAS – which leads to an increase in the hypertension level – increases the incidence of cancer, as well as the risk of cancer progression and mortality in cancer patients (2). Chronic inflammation and high Vascular Endothelial Growth Factor (VEGF) levels during hypertension contribute to endothelial dysfunction and angiogenesis, respectively, which may then be auxiliary factors during cancer development (5).

Cancer progression is a sequential process, beginning with the proliferation of cells at the site of origin and results in metastasis to distant sites in the body (6). Metastasis is the most critical process during cancer development, attributed to cancer-related deaths as the main cause. The veracity of this can be determined by the fact that most colon cancer deaths are related to liver metastasis (7). In hypertensive patients, the overstimulation of AT1R contributes to the vascular remodelling that supports tumour cell movement during metastasis (8). Furthermore, Ang II/AT1R signalling activates NF-κB, which promotes tumour growth and prevents apoptosis (9).

NF-κB is a transcription factor that is expressed in all cell types, and it plays a crucial role during both cancer progression and metastasis. It acts as a survival factor by promoting cancer progression and inhibiting apoptosis. It also controls metastasis by inducing adhesion molecules, such as E-selectin, that help disseminate cancer cells, in order for them to adhere to the vascular endothelial cells and subsequently enter the target site (10). Moreover, it promotes the Epithelial Mesenchymal Transition (EMT) process, where cancer cells lose their polarity, adhesion, and migrate to invade neighboring tissues (6, 11). The EMT process involves an increase in the production of Matrix Metalloproteinases (MMPs) – such as MMP-2 and MMP-9 – which act as degradative enzymes that support cancer invasion and migration by extracellular matrix (ECM) degradation. On the other hand, NF-κB promotes angiogenesis during cancer progression, through the upregulation of VEGF, which is responsible for neo-angiogenesis during both invasion and migration (2, 10). It is posited that NF-κB activity could be reduced by ACE inhibitors (ACEIs) and AT1R blockers (ARBs) (12).

ACEIs and ARBs are widely used as anti-hypertensive drugs. ACEIs reduce Ang II production, leading to the prevention of signalling in Ang II receptors; ARBs, meanwhile, block the AT1R signalling. They are suggested as an adjuvant therapy for cancer patients, but their effect as antitumour agents has not yet been agreed and confirmed, as they have conflicting effects on cancer (2). As demonstrated in **Table 1**, a vast proportion of the studies indicate the drugs’ efficacy in the regression of tumour development, while a limited number of studies found that ARBs such as losartan induce cancer progression, angiogenesis, and increase the risk of cancer – lung cancer, in particular (15, 33). One possible reason behind that confliction is that some ARBs, such as telmisartan, are able to activate the Peroxisome Proliferator-Activated Receptor-gamma’s (PPAR-γ) signalling, which controls the proliferation of cancer and promotes apoptosis. Other reasons to explain the confliction between studies could be the study design, the type of cancer involved in the study, the period of drug administration, or the intake of multiple medications by the patient (2, 34).

**Table 1:** The effect of some of ARBs and ACEIs treatments on the most common types of cancer.

Drug	Mode of action	Experimental model	Type of cancer	Pharmacological Effect	Reference
Enalapril and Aspirin	ACEI	Mice	Pancreatic cancer	- Inhibit cancer progression and invasive tumor formation.	(13)
Losartan	ARB	Human cell lines and tissues	EC	- Inhibit cell proliferation.	(14)
		Human	Lung cancer	- Increase the risk of cancer.	(15)
		Human	Melanoma	- Promote cell proliferation.	(16)
		Mice and human	Breast cancer	- Reduce tumor growth and angiogenesis.	(11, 17, 18)
		Mice	CRC	- Decrease angiogenesis. - Reduce lung metastasis.	(19)
		Mice	PC	- Inhibit tumor size and growth.	(20)
		Mice	Pancreatic Cancer	- Reduce desmoplasia. - Improve drug delivery. - Inhibit cancer progression. - Prolong survival.	(21, 22)
		Mice	Glioma	- Inhibit tumor growth and promote apoptosis.	(23)
Telmisartan	ARB	Human cell lines	PC	- Inhibit cell growth.	(24)
Olmesartan	ARB	Mice	Pancreatic cancer	- Inhibit cell proliferation and tumor growth.	(25, 26)
Captopril	ACEI	Mice	NSLC	- Reduce tumor volume. - Delay lymph node metastasis.	(9, 27)
		Mice	CRC	- Regress CRC liver metastasis.	(28)
		Mice	RCC	- Inhibit tumor growth. - Reduce tumor size.	(9, 29)
		Mice	RCC	- Promote tumor growth. - Decrease survival.	(30)
Candesartan	ARB	Mice	NSLC	- Reduce tumor volume. - Delay lymph node metastasis.	(27)
		Mice	Bladder cancer	- Inhibit tumor growth and angiogenesis.	(31)
		Mice and human	PC	- Reduce tumor volume. - Inhibit tumor angiogenesis.	(32)
		Mice and human	Breast cancer	- Inhibit tumor growth and angiogenesis	(18)
Perindopril	ACEI	Mice	Breast cancer	- Reduce tumor volume. - Decrease VEGF levels.	(9)

RAS is not only expressed systemically – in the liver, kidney, and lung – but is also expressed locally in different tissues – such as breast, pancreas, brain, ovaries, adipose, and heart tissue – where it is involved in tissue remodelling and endothelial dysfunction (1, 35, 36). Dysregulation of local RAS contributes to cancer metastasis, adhesion, invasion, angiogenesis, proliferation, and EMT. The exact role of each part of RAS is, however, contradictory depending on the type of cancer, the stage of cancer, the dose of, and interval between, the administration of ARBs and ACEIs, the tissue affected, and the expression level of Ang II receptors between cancers (2, 37). In the following review of literature, the aim was to clarify and summarise the effect of RAS components on the most common cancers.

## Breast Cancer

Breast cancer is a serious worldwide health problem, with high incidence and mortality rates among women. It is no longer confined to just older women; recent years have witnessed an increase, even amongst young women, of more aggressive phenotypes (38). Previous studies reported that RAS components are locally expressed in breast tissue and thus may play role in breast cancer pathology (39). In normal breast tissue, RAS activity mainly relies on the alternative pathway (ACE-2/Ang 1-7/MASR), whilst in breast cancer tissue, the classical pathway (ACE/Ang II/AT1R) was the prominent (40). So that, Ang II and its receptors merited to be therapeutically targeted by researchers using ARBs and ACEIs (41).

Ang II/AT1R axis plays a pivotal role in solid tumours. In breast cancer, it has four functional effects. First, it promotes tumour growth through the AKT and ERK1/2 signalling pathways (9, 42) and activation of the CARMA3-Bcl10-MALT1(CBM) signalling complex, which then induces NF-κB production (43). Secondly, it induces EMT through AKT phosphorylation (42) and the TGF-β/Smad signalling pathway, where the Snail1-Smad3/4 complex reduces E-cadherin (11). In addition, Oh et al. (11) revealed that the inhibition of smad4 in breast cancer cells – those that have high levels of AT1R – restored the E-cadherin level and reversed the epithelial mesenchymal phenotype (11). Thirdly, it supports invasion and angiogenesis through the induction of MMP-9, MMP-2, and VEGF expression, which plays a role in ECM modulation (11, 44). Fourth, it stimulates lymph node metastasis and cell migration through CXCR4/Sdf-1α signalling, which activates focal adhesion kinase (FAK)/Ras homolog gene family member A (RhoA) signalling. FAK/RhoA signalling directs cell movement, while CXCR4/Sdf-1α signalling helps tumour cells to reach lymph nodes (45).

Interestingly, the effect of CBM-dependent NF-κB activation is not only confined to tumour growth (46) but also extends to affect the tumour’s microenvironment by stimulating the secretion of VEGF, interleukin-6 (IL-6), IL-8, and IL-1B. VEGF contributes to angiogenesis, while IL-1B modulates immune tolerance to allow cancer metastasis (43).

In order to discern whether the functional effects of Ang II in breast cancer through AT1R or AT2R, Cambados et al. (42) demonstrated the effect of blocking AT1R or AT2R on Ang II induced AKT and ERK1/2 signalling pathways in breast cancer cells. Both AKT and ERK1/2 activity was significantly reduced after only AT1R blocking, indicating that the detrimental proliferative effect of Ang II in breast cancer was through AT1R (42). The Ang II level was high in breast cancer-related death, compared to that in unrelated breast cancer death, thus supporting Ang II’s role in breast cancer mortality. In addition, Ang II induces breast cancer angiogenesis, cell proliferation (47), and migration by stimulating the PI3K/AKT/NF-κB pathway through AT1R (48).

Previous studies illustrated that AT1R was highly overexpressed in breast cancer, therefore indicating its vital role in breast cancer growth and progression. Furthermore, Oh et al. (11) observed that AT1R function was supported by a high co-expression of X-linked inhibitor of apoptosis and poly (ADP-ribose) polymerase, which help cancer cells to evade apoptosis and promote cancer progression (11). The expression of AT1R was inversely correlated with the metastatic potential of breast cancer cell lines. Kowalska et al. (49) observed that MCF-7 has higher metastatic potential compared to the MDA-MB-231 and a lower AT1R level (49). Moreover, AT1R was expressed in breast cancer at a high clinical stage, and it was correlated with high cell proliferation and vascular density (39).

The role of AT2R in breast cancer pathophysiology remains unclear. Previous studies suggested that AT2R blocking may delay cancer progression (50). Arrieta et al. (39) found that AT2R expression was low in breast cancer cells compared to the normal breast cells, indicating that AT2R may not be associated with breast cancer cell proliferation (39). In a different study, however, breast cancer tissue showed higher AT2R levels compared to the normal tissue. This confliction in the functional effect and expression level of AT2R may be due to the functional association between AT2R and AT2R Interacting Proteins (ATIPs), as ATIPs increase AT2R availability by transporting it from the cytoplasm to the cell surface (51, 52).

The anticancer effect of Ang 1-7 through MASR has been proven by different studies where Ang 1-7 was able to inhibit fibrosis, reduce tumour weight and volume (40, 51), restore mesenchymal epithelial transition, as well as impede angiogenesis and metastasis induced by Ang II through inhibition of VEGF and MMP-9 expression (42). In particular, the presence of Ang 1-7 in breast cancer ameliorates the Ang II effect, but the Ang 1-7 and MASR expressions are low in breast cancer and continue decreasing with cancer progression (48). High ACE expression reduces overall survival in breast cancer patients, while high ACE-2 expression improves cancer prognosis and is associated with low metastatic potential of breast cancer (40).

There are many studies that have discussed the role and efficacy of using ARBs and ACEIs in cancer treatment. With regards to breast cancer, the majority of studies suggested that there is no association between use of ARBs or ACEIs and breast cancer risk (41), recurrence, and overall survival (51). Some studies reported that hypertensive patients are at low risk of breast cancer because they are using ACEI drugs that prevent Ang II formation (47). Although the use of ARBs and ACEIs in breast cancer treatment is debated, the inhibition of breast cancer growth (47), angiogenesis (53), and metastasis (50) were achieved by administrating AT1R blockers, which downregulate VEGF in human breast cancer cells (39) and inhibit NF-κB (46). Bakhtiari et al. (46) observed that the cytotoxic effect of Olmesartan with Bay 11-7082 on the MCF-7 breast cancer cell line was higher than treatment with Olmesartan alone. Olmesartan inhibits cancer cell growth by blocking AT1R, while Bay 11-7082 promotes cell apoptosis through NF-κB inhibition (46). Blocking AT1R with losartan reduced the CXCR4 expression on the MDA-MB-231 cell membrane and Sdf-1α in lymph nodes, greatly reducing the metastatic potential of breast cancer (15, 45).

Tamoxifen (TAM) is an Estrogen Receptor (ER) blocker that is used to treat all the stages of ER positive breast cancer. Despite its ability to reduce breast cancer recurrence and mortality, TAM resistance occurs in breast cancer patients after 5 years of TAM administration. Namazi et al. (50) tried to decipher the mechanism behind TAM resistance using an AT1R blocker (Losartan) and they found that AT1R was highly expressed in TAM-resisting MCF-7 cells compared to untreated MCF-7. They therefore suggested that blocking ER through use of TAM may promote an AT1R signalling pathway to maintain tumour growth and proliferation and, by using losartan, they observed a significant decrease in cell proliferation and noted that ER sensitivity was restored. They concluded that the combination treatment of ER positive breast cancer with losartan and TAM may prevent TAM resistance (50).

Recent studies suggest that the investigation of the tumour microenvironment provides an overview demonstrating how cancer cells interact with the surrounding environment, hence, providing better understanding and, as a result, an increase in possible therapeutic targets. Zhu et al. (54) explored the effect of combinational therapy on breast cancer using a nano carrier called Glycolipid-Based Polymeric Micelles (GLPM) to elucidate how angiotensin II type 1 receptor blockers modulate the tumour microenvironment. Telmisartan -ARB- loaded GLPM (GLPM/Tel) was administrated alone, followed by the combinational administration of GLPM/Tel and doxorubicin -apoptotic drug- loaded GLPM in the breast cancer tumour’s microenvironment. GLPM/Tel alone was able to improve drug distribution, suppress Cancer-Associated Fibroblasts (CAFs) by decreasing α-smooth muscle actin -markers of activated CAFs-, and destabilise stromal components by downregulation of connective tissue growth factors, while combinational therapy increased drug efficacy through the PPAR-γ pathway, as well as the uniformity of drug penetration due to the previous effect of the GLPM/Tel, administered alone, which relaxed blood vessels in the tumour’s microenvironment (54).

There are numerous factors that contribute to breast cancer etiology, including reproductive, environmental, and genetic factors (53). Genetic variations of RAS components (ACE and AT1R) affect their expression in breast tissue and, therefore, in cancer activity. Singh et al. (55) explored the effect of ACE insertion/deletion (I/D) and AGTR1 (A1166C) gene polymorphisms on a protein level, and they found that DD homozygote polymorphism of the ACE gene was associated with high levels of ACE and Ang II. Additionally, in Northern Indian women, the C allele of the AGTR1 gene is associated with high sensitivity to Ang II (55). Moreover, interactions between genes may be implicated in the risk of breast cancer: in Iranian women, it was observed that the interaction between AGTR1 gene polymorphism and ACE gene polymorphism increased the risk of breast cancer, whereas, in Chinese women, the same interactions were not associated with breast cancer risk (53).

On the other hand, a previous study revealed that the AGTR1 gene polymorphism is not associated with the risk of breast cancer in Brazilian and Iranian women, while it is significantly associated with breast cancer in Caucasian women (53). Thus, we can conclude that the polymorphisms of ACE and AGTR1 genes could be considered as a risk factor of breast cancer, taking into consideration racial disparity (**Table 2**) (55).

**Table 2:** The effect of genetic variation of ACE and AGTR1 genes on breast cancer in different ethnicity (53, 55).

Country/Population	Gene Polymorphism	Breast cancer association
Mexican women	D allele of ACE gene	Increase risk of breast cancer
Mexican and Caucasian women	C allele of AGTR1 gene	Decrease risk of breast cancer
Chinese women	AA (A240T) and insertion homozygote (II) of ACE gene	Decrease risk of breast cancer
Egyptian postmenopausal women	C allele of AGTR1 gene	Increase risk of breast cancer
Kashmiri women	Insertion homozygote (II) of ACE gene	Increase risk of breast cancer
	ACE heterozygote (ID) genotype	Decrease risk of breast cancer
Ukrainian women	AC genotype of AGTR1 and D allele of ACE gene	Increase risk of breast cancer
North Indian women	AC, CC (A1166C) and C allele of AGTR1 Deletion homozygote (DD) and D allele of ACE gene	Increase risk of breast cancer
	Insertion homozygote (II) of ACE gene	Decrease risk of breast cancer

## Gynecologic Cancers

### Ovarian Cancer

Amongst the group of gynecological cancers, ovarian cancer has the highest mortality rate due to its ambiguous and nonspecific symptoms (56). Ovarian cancer patients have short overall survival rates as this type of cancer often has a worse prognosis related to the lack of distinct validated markers that can help provide accurate diagnoses at early cancer stages. RAS was believed to be expressed locally in ovarian tissue, and increases in the expression of its components – Ang II and AT1R – were found to be correlated with neoplastic development of ovarian tissue (13).

Considered the most aggressive type of gynecological cancers, the metastasis process of ovarian cancer predominantly occurs due to the formation of multicellular spheroids (MCS), which help disseminate cells to aggregate with each other, avoiding cell death and promoting cancer metastasis. AT1R is highly expressed in ovarian cancer and known to promote both EMT and metastasis to the peritoneal cavity, leading to increasingly negative outcomes. Ang II/AT1R signals promote cancer development and progression, causing them to emerge as potential targets in the treatment of ovarian cancer. Zhang et al. (57) found that Ang II was overexpressed in ovarian cancer, stimulating AGT gene expression to produce more Ang II. This vicious circle therefore leads to the progression of cancer and the formation of MCS. Ang II/AT1R signalling activates the ERK and PI3K/AKT pathways, inducing lipogenesis through the activation of the Sterol Regulatory Element-Binding Protein (SREBP) pathway. The SREBP pathway upregulates lipogenesis enzymes, including Stearoyl-CoA Desaturase-1 (SCD1), an endoplasmic reticulum enzyme responsible for lipid desaturation. Cancer cells tend to avoid apoptosis through lipid hemostasis, and reduction of endoplasmic reticulum stress by activation of the lipid desaturation process, thus relieving endoplasmic reticulum stress caused by saturated fatty acid overload. Furthermore, the effect of Ang II is not limited to AT1R but can also be converted to Ang 1-7 in the presence of losartan -AT1R blocker-, which downregulates AT1R. In a recent study, the co-treatment of ovarian cancer with Ang II and losartan was able to reduce tumour size and increase cell death, which was reduced in the presence of Ang II alone. SCD1, which helps cancer cells to avoid cell death, was not expressed in the presence of losartan. Therefore, it was concluded that, in the absence of the AT1R effect, Ang II could be converted to Ang 1-7, acting through MASR to reduce cancer metastasis (57).

Beyazit et al. (13) found that ACE was highly expressed in ovarian cancer patients, regardless of the cancer stage, and suggested ACE as a therapeutic target for future studies (13). Previous study found that ATIPs that had a tumour suppressing effect were downregulated in ovarian cancer cells, while the high-mobility group, AT-hook 2 (HMGA2), was overexpressed. In epithelial carcinoma, HMGA2 promotes tumour growth and metastasis through activation of the ERK signalling pathway and EMT phenotype, respectively. Ping et al. (58) investigated the effect of ATIP3a induction in ovarian cancer, where they found that the HMGA2 mediated tumour growth, migration and invasion were attenuated, and the HMGA2 level was downregulated. The ATIP3a, therefore, could be considered a viable candidate for ovarian cancer therapy (58). Interestingly, Cho et al. (59) suggested that ARBs were the most efficient type of antihypertensive drugs in the reduction ovarian cancer recurrence rate, as ovarian cancer tissue has high concentrations of Ang II receptors (59).

### Uterine Cancer

Uterine cancer is the fourth most common cancer among U.S. women. It is classified based on the affected tissue into endometrial carcinoma, other carcinomas, carcinosarcoma, and sarcoma (60). RAS components are well-known to be expressed normally in the uterus during the menstrual cycle, where Ang II is required to induce VEGF production during the formation of follicles, building a new endometrium, and the development of oocytes. VEGF is the main regulator of blood vessel formation and it is overexpressed in Endometrial Cancer (EC) compared to the normal people. Additionally, the RAS components were found to be dysregulated in EC, where cancer progression requires Ang II mediated VEGF production. Estrogen/AT1R signalling was shown to promote estrogen receptor proliferation and, as a result of that, AT1R was highly expressed in primary stages when compared to the advanced stages of EC. In addition, inhibition of VEGF was able to reduce EC proliferation and migration through a small interfering RNA and Ang II degrading enzymes, respectively (14).

In terms of Ang II’s effect on EC cell lines, it promotes cell proliferation, survival, EMT phenotype, and migration in a dose-dependent manner, where the amount of Ang II needed to exert its tumorigenic effect increases as the EC progresses or loses the differentiated phenotype. Ang II/AT1R also supports migratory and invasive properties through the upregulation of EMT-related genes and the downregulation of E-cadherin – a cell adhesion gene – in the advanced stages compared to the early stages. TGF-β is known to have a contradictory effect: in the early stages of cancer, it exerts a tumour suppressor effect, while at the later stages, it promotes cancer progression. Nowakowska et al. (61) reported low expression of TGF- β at the early stages of EC and high expression at advanced stages, with expression regulated by Ang II. Interestingly, AT1R blocking had a slight effect on Ang II-associated EC development, indicating that the effect of Ang II could be adapted to another signalling pathway, in the absence of AT1R (61, 62).

The tumour suppressing effect of AT2R in uterine leiomyosarcoma was investigated by LÜtzen et al. (63), where the selective blocking of AT1R in quiescent uterus cells promoted cell differentiation and apoptosis through Ang II/AT2R-mediated PPAR-γ signalling. AT2R increased the expression of Calponin and Smooth Muscle Protein 22α – differentiation markers – reducing cell proliferation in uterine leiomyosarcoma. In addition, AT2R interacted with ATIPs, activating downstream apoptosis by binding with PPAR-γ (63).

The genetic variations of AGT and AGTR1 genes in Australian women were investigated by Pringle et al. (64). They identified that the G allele of the AGT gene was associated with a high expression of AGT, whilst the C allele of the AGTR1 gene was associated with an increased AT1R level. In EC, AGT overexpression was associated with the anti-angiogenic effect of RAS, while high levels of AT1R was associated with cancer growth and progression. Interestingly, the C allele of the AGTR1 gene was predominant in Australian women with EC, but not in the G allele of the AGT gene, which indicates the role of gene polymorphism in EC (64).

## Prostate Cancer

Prostate cancer (PC) is the most common male cancer and it has two types: androgen-dependent PC and androgen-independent PC (65). RAS was previously known to be expressed in normal prostate tissue, as well as cancer cells, reflecting its influential role in normal cell functioning and malignant transformation. In normal prostate cells, it contributes to spermiogenesis, sperm motility, and sperm survival. Domińska et al. (66) noticed that the long exposure of normal prostate cells to Ang II affects cell morphology, enhances cell proliferation and survival through upregulation of BCL2/BAX ratios, and promotes ECM degradation through the increase of MMP-2 and MMP-9 production. All the above-mentioned effects indicate that the dysregulation of Ang II expression in the prostate increases the risk of PC (66). It was found that Ang II upregulates survivin – a PC prognostic marker – activating the IGFR1/AKT pathway in PC cells. Activation of IGFR1/AKT signalling in androgen-dependent prostate cancer cells resulted in androgen resistance. Moreover, Ang II was able to enhance PC progression and invasiveness, by increasing MMPs production (67).

Ang II receptors were found to be overexpressed at the early stages of PC (67). In addition, Kowalska et al. (49) revealed that there is a positive correlation between AT1R expression and the metastatic potential of PC cells (49). While *in vivo*, the overexpression of AT2R in PC was found to inhibit tumour growth, reduce Ki-67 – associated with PC aggressiveness – expression, and induce apoptosis. In addition, AT2R expression was inversely correlated with the degree of PC aggressiveness (68). Inhibition of AT1R, and stimulation of AT2R, were suggested as potential therapeutic targets for PC treatment (20). The effects of AT2R stimulation was investigated by Ito et al. (69), who identified that *in vivo* and vitro stimulation of AT2R by compound 21 – AT2R agonist – stimulates cell apoptosis and decreases the expression of androgen receptors, thus reducing cell proliferation. Therefore, AT2R stimulators were suggested to be human PC candidates in chemo-preventive therapy (69).

Long-term use of antihypertensive drugs (ARBs or ACEI) have been found to reduce the risk of PC among hypertensive patients (70). Additionally, using ARBs at high concentrations (100-400µM) were observed to increase PC cell death *via* promoting the cancer cell autophagy represented by the presence of microtubule-associated protein 1A/1B-light chain 3-positive foci, and an increase in autophagy-related gene expression. In addition to previous ARBs effects, fimasartan in particular was able to reduce PC migration, thus it may prove to be a promising therapeutic agent for PC in hypertensive patients (71).

Furthermore, NFκB expression increases the metastatic potential and drug resistance in PC, and it has been found to be correlated with advanced stages of cancer, while androgen receptor expression was found to be inversely correlated, as prostate cancer becomes androgen-independent in the late stages. The expression of the NFκB family and androgen receptors were found to be affected by Ang II during PC development (72). In androgen-independent PC, Ang II/AT2R and Ang 1-7 were found to reduce Protein Tyrosine Kinase (PTK) activity. PTKs promote PC progression in advanced stages, where they were found to be upregulated. Therefore, the presence of Ang II and Ang 1-7 were suggested to be crucial in the advanced stages of PC or in androgen independent PC, to provide a protective effect. As a result, ACEIs were recommended to be administrated carefully, as their effect may not be always against cancer growth (73).

## Renal Cancers

Hypertension has been considered as a renal cancer risk factor, as they share mutual Ang II- mediated signalling pathways – such as the pathways that are involved in the induction of proliferation and the inhibition of apoptosis. In addition, overactivation of RAS induces oxidative stress, as well as DNA damage and mutation, increasing the susceptibility of developing kidney cancer, with an accumulation of mutations in the rat treated with 400µg Ang II/kg per day for 20 weeks (74).

Renal cell carcinoma (RCC) is a kidney cancer that is mostly detected after distant metastasis and eventually results in nephrectomy, due to the lack of effect by conventional therapy. Furthermore, it has a high recurrence rate after surgery. These concerns promoted researchers to identify prognostic markers and, from this, possible therapeutic targets to improve cancer prognosis and treatment (75, 76).

AT1R and MASR were found to be upregulated in bladder cancer, while AT2R was downregulated. The effect of AT2R stimulation in bladder cancer was demonstrated by Pai et al. (70), where the tumour growth and angiogenesis inhibited through inactivation of the ERK pathway, and VEGF production reduced, respectively. Moreover, AT2R stimulation induces apoptotic signalling pathways, including P38 MAPK, caspase-3, and caspase-8 (77). Furthermore, AGTR2 expression were able to increase the overall survival of bladder cancer patients (78).

Ang II/AT1R arm was found to be overexpressed in RCC and to promote cell growth, proliferation, and lung metastasis through upregulation of VEGF – an angiogenic factor – and stimulation of CD43, an inflammatory cytokine (75, 76). In contrast to the well-known anti-tumour and protective effect of Ang 1-7/MASR signalling in many tumors, Ang 1-7/MASR signalling was found to stimulate RCC by increasing the migration ability in clear cell carcinoma and renal cell adenocarcinoma (76).

In addition, Araújo et al. (75) found that the use of ARBs or ACEIs alone was able to reduce renal cancer growth and lung metastasis *via* downregulation of VEGF, while combinational use of ARBs and ACEIs was less effective. Combinational treatment may prove to be less efficient, as the inhibition of AT1R and Ang II production at the same time increases the bradykinin production – which promotes cancer growth – inhibiting the Ang II/AT2R-mediated tumour suppression effect and allowing renin production, which promotes Ang II-independent growth signalling pathways. Thus, the overall effect of combinational treatment could support the tumourigenic effect more than antitumourigenic effect (75, 76).

In contrast to the previous study, Xie et al. (33) reported that long-term use of ARBs and ACEIs increased the risk of kidney cancer, and only ARB was correlated with the risk of bladder cancer. In addition, other studies indicated that there was no association between antihypertensive drugs and the risk of kidney and bladder cancer. These contradictory observations might be due to the degree of hypertension in the patient, and both the time and dose of antihypertensive drug administration (33).

Interestingly, the combinational treatment of kidney cancer with Sunitinib – a receptor tyrosine kinase inhibitor – and Captopril – ACEI – was able to reduce cell viability, which had not been obtained in each of them alone (76). In case of bladder cancer, ACEIs and ARBs were able to increase the chances of recurrence-free survival after immunotherapy, but the mechanism behind this remains unknown (79). Bladder cancer also has resistance against conventional therapy, although it is mostly non-invasive and could be removed by a cystectomy, but the problem remains due to the fact that it will recur in 75% of patients after a specific period of time (77, 80).

## Gastrointestinal Cancers

### 1-Liver Cancer

Liver cancer is the second leading cause of cancer-related death worldwide (81), with hepatocellular carcinoma (HCC) and intrahepatic cholangiocarcinoma being the most common types of liver cancer (82). RAS plays a pivotal role in the angiogenesis of normal and abnormal liver tissue. Understanding the regulation of the angiogenesis during HCC development helps to improve cancer diagnosis (83). Due to the high recurrence rate of liver cancer after surgical removal with more aggressive potential, the need arises to find adjuvant therapy that increases disease-free survival, and overall survival, among liver cancer patients (84). Ye et al. (83) compared the level of RAS components (Ang II, Ang 1-7, and ACE-2) during abnormal development of liver disease, from fibrosis to HCC. They found that as the disease severity increased, Ang II, Ang 1-7, VEGF, and CD34 are upregulated, while ACE-2 levels are downregulated. They therefore revealed that RAS is critical for HCC progression and angiogenesis, and the reduction of ACE-2 might be correlated with HCC poor prognosis (83).

In a later study conducted by Liu et al. (85), Ang II was shown to upregulate AT1R and stimulate cell proliferation in HCC, by increasing proinflammatory cytokines and nicotinamide adenine dinucleotide phosphate oxidase activity, which increases Reactive Oxygen Species (ROS) level (86), while Qi et al. (87) found that Ang II promotes EMT, migration, and invasion through activation of the TGF-β signalling pathway in HCC (87).

AT1R was found to be overexpressed in liver cancer and to promote Ang II-mediated angiogenesis and fibrosis through the upregulation of VEGF-A, TGF-β, and increasing microvascular density (37, 84, 88). Sartans – ARBs – in addition to being nontoxic and safe with regards to long-term use, were suggested by Facciorusso et al. (84) as potential therapeutic agents due to their efficacy in prolonging survival, as well as in reducing the recurrence rate after surgical resection of liver cancer (84). Moreover, the effect of Candesartan – ARB – was estimated by Fan et al. (88), enabling the attenuation of Ang II/AT1R/VEGF mediated angiogenesis, metastasis, and tumour growth in liver cancer at dose of 2 and 10 mg/kg/day (88). Telmisartan – another ARB – was found to reduce cell proliferation through activation of the AMP-activated protein kinase (AMPK) alongside inhibition of the mammalian Target of Rapamycin (mTOR) signalling pathways, which inhibit cell cycle regulatory proteins such as cyclin D1 and cyclin E, thus arresting the cell cycle in poorly differentiated HCC cells (89). Furthermore, Feng et al. (90) found that Irbesartan – ARB – effectively attenuated VCAM-1-mediated HCC cells adhesion during lung metastasis through inhibition of Ang II/AT1R-activated P38/MAPK pathway which responsible for increased VCAM-1 expression during HCC metastasis (90).

On the other hand, combinational treatment of HCC with azilsartan – an ARB – and Bay11-7082 – an NFκB antagonist – was found to induce apoptosis pathways by elevating ROS, thus releasing cytochrome c from the mitochondria into the cytosol and reducing the Bcl-2/Bax ratio in HCC (25). Another study found that losartan as an ARB supported Lenvatinib anticancer effect in the liver cancer through inhibition of Ang II-mediated cancer cell growth and increasing cell apoptosis. Besides that, losartan as an adjuvant therapy showed an antiangiogenic activity by reducing VEGF-A production induced by Ang II and Lenvatinib (91). In addition to the ARBs, the effects of ACEIs on HCC were evaluated by Saber et al. (92), leading them to conclude that ARBs and ACEIs have the potential to trigger HCC regression and angiogenesis suppression in mice models treated with diethylnitrosamine (92).

Ang 1-7 was known to play an antitumorigenic role in the majority of cancerous tissues. Its effect on HCC in mouse models was investigated by Liu et al. (37), where cell proliferation and angiogenesis were inhibited, and cell apoptosis was activated, following Ang 1-7 treatment in a dose and time-dependent manner (37). The antiproliferative effect of Ang 1-7 was demonstrated by the low expression of AT1R and the high expression of AT2R and MASR. Upregulation of AT2R was suggested to promote apoptosis by increasing caspase-3 and p38- MAPK activity. Interestingly, the anticancer effects of Ang 1-7 are not only mediated by MASR, but AT2R partially contributes to growth inhibition and the apoptosis induction effect excreted by Ang 1-7 in HCC (37). Moreover, the antiangiogenic effect of Ang 1-7 was evidenced by decreasing vascular density and VEGF-A expression. The possible mechanisms by which Ang 1-7 decreases VEGF-A expression in HCC was suggested to be, the inhibition of ERK signalling, in addition to the downregulation of hypoxia inducible factor-1α and cyclooxygenase 2 (37).

### Colorectal Cancer

Colorectal Cancer (CRC) is one of the most common cancers among both men and women. Age, sex, nutrition, genetic background, and obesity are considered as CRC risk factors (93). RAS components play an important role in the normal physiological function of the colon, where the Ang II – through AT1R and AT2R – stimulates sodium and water absorption and secretion, respectively. It was found that RAS components are dysregulated in CRC, which indicates the contribution of RAS in CRC pathology. CRC predominantly metastasizes to the liver, where the angiotensinogen production is normal, leading to the notion that RAS also plays a pivotal role in CRC metastasis. In CRC liver metastasis, ACE, Ang II, and MASR were found to be upregulated, while AT1R noted as being downregulated in comparison to the normal liver tissue. ACE converts the angiotensinogen produced by the liver into Ang II, which, through AT1R, stimulates angiogenesis and metastasis through the upregulation of VEGF and TGF-β, respectively. Although AT1R downregulated in the liver during CRC metastasis, Kupffer cells (KCs) showed overexpression of AT1R, which was posited as being responsible for promoting CRC liver metastasis (94, 95). Shimizu et al. (95) confirmed the previous suggestion by investigating the effect of AT1R knockout in CRC mice models, where they found that liver metastasis was suppressed and TGF-β production was downregulated in KCs, thus indicating that liver metastasis is promoted by KCs, stimulating TGF-β production through AT1R signalling (95).

RAS inhibitors are associated with decreased risk and mortality of CRC (96). ACEIs and ARBs treatments were found to increase recurrence-free survival in the early stages of CRC and left-sided CRC cases. Conversely, the overexpression of the AGTR1 gene – which was observed in the advanced stages – was associated with poor recurrence-free survival (97). In addition, ARBs were able to increase overall survival as well as progression-free survival (PFS) in patients with metastatic CRC who underwent first line chemotherapy (98).

Moreover, activating protein-1 (AP-1) complex was proved to initiate CRC. The administration of Irbesartan – an ARB – were found to inhibit the AP-1 pathway by inhibiting JUN gene expression, which encodes AP-1 (99). Ruderman et al. (100) observed the inhibitory effect of losartan on colorectal cancer mice models by downregulating VEGF protein, decreasing the number of tumours, and abolishing changes in blood supply during neoplastic angiogenic transformation (100). While in case of Captopril and Irbesartan were found to reduce tumour size by supporting the antitumour effect of KCs in CRC liver metastasis. Moreover, captopril was found to downregulate ACE expression, thus inhibiting Ang II production and promoting Ang 1-7 production, which plays an antiproliferative role through the MASR in CRC liver metastasis (94).

### Pancreatic Cancer

Pancreatic cancer is expected to be the second leading cause of death among cancer patients in the United States by 2030. Pancreatic Ductal Adenocarcinoma (PDAC) and pancreatic endocrine tumours are the main types of pancreatic cancer. Although it can be treated by chemotherapy and/or a pancreatectomy, eventually it will recur and develop. Additionally, its ambiguous, refractory nature and the absence of early diagnostic markers are considered to be obstacles towards better outcomes and treatment. RAS components were suggested to be novel biomarkers or therapeutic targets in some cancers. In PDAC, Ang II was found to promote desmoplastic reaction and cell proliferation by activation of the protein kinase C (PKC) and ERK signalling pathways, which promote pancreatic stellate cells to overproduce TGF-β. TGF-β overproduction contributes to the desmoplastic reaction (101, 102).

Ang II receptors are known to be expressed locally in both normal and cancerous pancreas tissue. AT1R was found to be highly expressed in pancreatic cancer, while AT2R exhibited low expression compared to the normal surrounding tissue. AT2R agonists emerged as potential therapeutic agents for PDAC, as they were able to inhibit cell growth and induce cell apoptosis in AT2R-expressing PDAC cells (103).

In the case of AT1R, its overexpression was found to promote pancreatic cancer progression by interfering with the antitumorigenic role of MicroRNA 410 (miR-410), which inhibits cell proliferation, angiogenesis, and invasion. Moreover, miR-140 was found to be downregulated in cancer cells compared to the surrounding normal tissue, indicating AT1R’s oncogenic role in pancreatic cancer (104). Therefore, based on previous studies, therapeutic strategies that involve AT2R agonism and AT1R antagonism are recommended for better pancreatic cancer outcomes.

The use of ARBs was shown to decrease the risk of death and increase overall survival in pancreatic cancer patients who underwent surgical removal (105). Losartan was demonstrated as being able to inhibit cancer progression and prolong survival in pancreatic cancer (21).

### Esophagus Cancer

Esophageal Squamous Cell Carcinoma (ESCC) and Esophageal Adenocarcinoma (EAC) are types of esophagus cancer, with ESCC being the most common type among esophagus cancer patients (106, 107). Although it can be treated, its overall prognosis remains unsatisfactory (106). RAS was previously suggested to be associated with hallmarks of cancer. Ang II *via* AT1R was suggested to be involved in Barrett’s esophagus transformation into EAC, as it had a regulatory effect on EAC development related proteins (108).

AT1R was found to be overexpress at high tumor stages and associated with low overall survival and poor prognosis. Moreover, Ang II/AT1R signaling was suggested to promote ESCC progression through mTOR activation in dose-dependent manner. The inhibition of AT1R either by AT1R blockers or siRNA were found to reduce cell proliferation in ESCC (106). In another studies, RAS inhibitors such as Captopril, Losartan, and Irbesartan showed improvement in the overall survival, inhibition of cell proliferation, and suppression of neovascularisation by decreasing VEGF production in ESCC, with each effect being dose-dependent. RAS inhibitors were therefore recommended as salvage therapy for ESCC (109).

Matsui et al. (107) investigated the effect of Telmisartan – ARB – on ESCC *in vivo* and *vitro*. They reported that the telmisartan did not affect apoptosis, but it was able to inhibit cell proliferation by reducing cyclin A2 and cyclin-dependent kinase 2 expression, which led to cell cycle arrest at the S-phase (107). With regards to EAC, telmisartan illustrated an antiproliferative effect through cell cycle arrest at the G0/G1 transition by reducing cyclin E and cyclin D1, and suppressing cell cycle regulatory proteins through the AMPK/mTOR signalling pathway. Therefore, telmisartan is suggested to be a potent antiproliferative therapy for esophagus cancer (110).

## Lung Cancer

Lung cancer is a complicated disease, consisting of several types of cancer, including adenocarcinomas, small cell lung cancer, and non-small cell lung cancer (NSCLC) (111). It has a low survival rate and a high mortality rate due to the propensity for late diagnosis at advanced stages of the disease, with the early stages being asymptomatic (112, 113). RAS components are known to be expressed in lung tissue and to contribute to lung cancer pathology (114).

Cancer Stem Cells (CSC) are immortal cells present in the tumour’s microenvironment. They are thought to mediate both cancer metastasis and its aggressiveness and are implicated in the tumour’s resistance against conventional therapy. Cell surface expression of CD133, CD44, and CD24 were determined as CSC markers (115). Ang II was found to promote cell proliferation and stimulate CSC-like phenotypes by increasing the CD133 expression in lung cancer cells (27). Interestingly, K. Yang et al. (116) revealed that, in the human NSCLC cell, Ang II/AT1R signalling was able to suppress anti-tumour immunity through the activation of programmed death ligand-1 expression, which inhibits T-cell activity in the tumour microenvironment (116).

Ang II was found to promote lung cancer progression through Epidermal Growth Factor Receptor (EGFR) transactivation, which activates the MEK/ERK pathway, or through AT1R activation, which upregulates micro RNA-21 (miRNA-21) – an oncogene – to stimulate the PI3K/AKT pathway through phosphatase and tensin homolog – a tumour suppressor – inhibition. In addition, miRNA-21 was found to be associated with short disease-free survival and short overall survival in NSCLC patients, subsequently leading to the suggestion that utilising the silencing of miRNA-21 as a potential lung cancer therapy needs more investigation (117).

Furthermore, EGFR was previously ascertained as promoting cancer development through the activation of the Mitogen-Activated Protein Kinase/Signal Transducer and Activator of Transcription (MAPK/STAT) signalling pathway, leading to EGFR tyrosine kinase inhibitors (TKIs) being administrated as an anticancer drug agent. Interestingly, NSCLC patients who are using TKIs were found to have longer PFS after ACEIs/ARBs treatment (114). In addition, telmisartan – ARB – treatment was shown to inhibit tumour growth, invasion, and migration in NSCLC, and to activate NSCLC apoptosis through inhibition of the PI3K/AKT signalling pathway (112). In a retrospective study of Korean cohort with median follow-up time 7.8 years, ARBs were found to decrease the risk of lung cancer compared with calcium channel blockers use among hypertension patients (118).

NSCLC constitutes the majority of lung cancer cases, with a low overall survival rate due to acquired drug resistance during treatment (112). In platinum-resistant NSCLC, AT1R, ACE, and VEGF were found to be upregulated, while ACE2 was downregulated. Following stimulation of ACE2 expression, tumour growth was inhibited and the production of AT1R, ACE, and VEGF were reduced, due to ACE2 being known to convert Ang II to Ang 1-7, which has been proven previously to reduce tumour growth, invasion, and angiogenesis through reduced VEGF production in NSCLC. Thus, ACE2 was suggested as an antiproliferative and antiangiogenic agent after the development of platinum-resistance in NSCLC (119). Moreover, most recent study conducted by Geng et al. (120) found that Ang 1-7/MasR downstream signalling play role in platinum-resistance NSCLC through inhibition of cancer growth and angiogenesis (120).

On the other hand, Tumour Necrosis Factor-Related Apoptosis-Inducing Ligand (TRAIL) resistance is an emerging concern in NSCLC cancer therapy, as TRAIL induces cancer cell apoptosis. One of the suggested mechanisms of TRAIL resistance is the autophagy process, which helps cancer cells to survive under stress conditions, such as chemotherapy. Combinational therapy of TRAIL treatment with candesartan – an ARB – was able to re-sensitise TRAIL, inhibit autophagy, and promote programmed cell death (121).

AT2R stimulation was shown to promote lung cancer apoptosis (27), leading to the suggestion of using AT2R plasmid in lung cancer gene therapy by Alhakamy et al. (122) and Ishiguro et al. (123). This is because AT2R overexpression was demonstrated to successfully and safely reduce lung cancer growth by apoptosis induction (122, 123). In addition, the inhibition of AT1R and stimulation of AT2R were suggested to be lung cancer therapeutic targets. A multi-walled carbon nanotube was used as a nano co-delivery system, complexed with the vector carrying AGTR2 gene and candesartan – an AT1R blocker – to investigate the effect of AT2R overexpression and AT1R blocking in lung cancer. This was observed by the potent inhibition of tumour growth and prevention of new blood vessels forming (124).

NSCLC is divided into Lung Adenocarcinoma and Lung Squamous Cell Carcinoma (LSCC). AGTR1 promoter, methylation, was found to be significantly high in LSCC compared to the normal surrounding tissue, prompting CHEN et al. (125) to suggest AGTR1 hypermethylation as an LSCC biomarker (125).

In contrast to aforementioned beneficial effects of ACEIs and ARBs in lung cancer, Kristensen et al. (126) found a positive association between the administration of high doses of ACEIs and the risk of lung cancer among hypertensive danish population (126). ACEIs administration could induce bradykinin accumulation which induce cancer proliferation and migration through B2 receptors (127). Another case-control study revealed that long term use of ACEIs and ARBs at high doses was associated with an increased risk of adenocarcinoma but not LSCC or small cell lung carcinoma (128). This contradiction in the studies’ findings could be explained by presence of confounding factors such as age, smoking, sex, obesity, alcohol consumption, and genetic background which could create bias in the final outcomes in relation to the lung cancer. However, the observed protective effect of ACEIs and ARBs against cancer mortality such in Brugts et al. (129) study and other studies cannot be neglected.

## Skin Cancer

Skin cancer is chiefly divided into melanoma and non-melanoma. Non-melanoma skin cancer is further subdivided into squamous cell carcinoma and basal cell carcinoma. Although melanoma is less common compared to non-melanoma types, it is the most lethal of the two (130). Moreover, it is an aggressive skin cancer that metastasizes predominantly to the lung, with lung metastasis positively correlated with short overall survival of melanoma patients. AT1R was found to be expressed in melanoma and in Myeloid Derived Suppressor Cells (MDSC) of pulmonary metastasis in mice models. MDSC are hematopoietic cells known to play an immunosuppressive role in the tumour’s microenvironment. Andrade et al. (2015) found that the number of MDSCs were reduced and the AT1R downregulated in lung metastasis mice models that had been treated with M1 – a homeopathic medicine. This led to the conclusion that the inhibition of melanoma growth and metastasis to the lung may partially be achieved by suppressing AT1R (131, 132).

The complex interaction of RAS components was found to play a critical role in skin cancer. Ang II was found to increase cell proliferation and invasion by increasing MMPs production and activity in human melanoma, and that effect was suggested to be through AT2R. On the other hand, N^+^/H^+^ Exchanger Isoform1 (NEH1) activity promotes cell motility in skin cancer. The interplay between RAS components and NEH1 activity in human melanoma was demonstrated by Olschewski et al. (133), where they identified that Ang II stimulates NEH1 activity and cell proliferation through the Ca^+2^/calmodulin signalling pathway, and that the effect was abolished by losartan – an AT1R blocker (133).

Additionally, Ang II/AT1R promotes pulmonary metastasis in melanoma by increasing E-selectin expression, helping in the adherence of melanoma cells to the lung during the endothelium adhesion stage of metastasis process. In an AGTR1 gene-lacking melanoma mice model, Ang II treatment did not affect cell proliferation, and the pulmonary metastasis was suppressed (134).

In a different study, Ang II was able to reduce cell migration by increasing adhesion, contraction, and the spheroid morphology of human melanoma cells, which helps to prevent the spreading of cancer cells. Furthermore, the blocking of AT1R supports the antimigratory effect of Ang II, while AT2R blocking had no effect (133).

In contrast to the previous studies, Renziehausen et al. (16) concluded that AT1R have an Ang II-independent antitumorigenic effect, while AT2R promotes cancer growth in an Ang II-dependent manner in melanoma cancer cells. They also found that the suppression of AT1R, either by losartan treatment or by shRNA, resulted in the growth promotion of AT1R-expressing melanoma cells. In addition, ectopic expression of the AGTR1 gene in melanoma cell lines led to cell death, while the loss of AT1R was suggested to be associated with a melanoma-aggressive phenotype. With regards to AT2R, Ang II treatment promotes cell proliferation in AGTR1 gene-lacking melanoma cells but not in AGTR1 and AGTR2 genes lacking melanoma cells, which proves the Ang II-dependent proliferative effect of AT2R. Moreover, AT2R agonist induces cell proliferation, while AT2R antagonist decreases melanoma cell growth (16).

In non-melanoma skin cancer, AT1R was found to be overexpressed in both head and neck squamous cell carcinoma (HNSCC). Ang II/AT1R signalling was found to stimulate the motility and invasion ability of HNSCC, while Ang 1-7 inhibits that effect only in the presence of Ang II. AT1R blockers and ACEIs were able to inhibit HNSCC invasion and growth, respectively (135). Recently Drucker et al. (136) revealed that there is no association between ARBs or ACEIs administration and the risk of skin cancer in people older than 65 years (136). In terms of genetic variations, low expressions of ACE and AGT genes are suggested to be associated with a low risk of basal cell carcinoma (137).

## The Effect of Urotensin II (UII) in the Common Types of Cancer

A major cellular homeostasis component, urotensin II (UII) contributes to the development of both acute and chronic diseases, inflammation, liver cirrhosis, and other conditions (138). It has been determined to affect angiogenesis and mitogenesis, using its receptor (UII-R) to exert a potent angiogenic effect *in vivo* as well as *in vitro* (139). Furthermore, according to research evidence, UII vasoconstriction and vascular impairment are underpinned by the Ang II pathway, as vascular responses to UII are inhibited when the angiotensin-converting enzyme pathway is suppressed. At the same time, Ang II seems to worsen UII-induced endothelial dysfunction (140). A noteworthy observation from a rat model study is that UII and Ang II exhibit synergistic action when administered together in the aorta, eliciting a strong contractile effect accompanied by heightened activity of PKC and phosphorylation of PKC-α/βII and myosin light chain (141).

### Breast Cancer

A number of tumor cell lines have been found to express UII and UII-R. When UII activates UII-R, extremely complex downstream signalling pathways are activated (142). A correlation has been proposed to exist between UII and UII-R expression and both menopausal status and extra-nodal and lymphatic invasion in the context of breast cancer tissue (139). Furthermore, the biology of breast cancer tumor seems to depend greatly on the UII protein, since breast cancer patients have been observed to have markedly lower plasma levels of this protein. Additionally, the likelihood of breast cancer development may be promoted by Thr21Met polymorphism in the UTS2 gene possibly through its influence on the molecular mechanisms underpinning disease pathogenesis (143).

### Prostate Cancer

During the invasion of prostate cancer cells, UII-R expression downregulation has a substantial inhibitory effect on cells motility that paralleled by reduction in the expression of CD61 and CD11a integrin as well as phosphorylation of FAK tyrosine. Hence, UII-R may serve as a marker of prognosis for human prostate adenocarcinoma (144). Furthermore, Gleason score upstaging and upgrading in cancer cases have significant ramifications for treatment. Biopsy and radical prostatectomy samples have been reported to have decreased tumor expression of UII-R when the Gleason score and clinical stage of prostate cancer were higher (145). On the other hand, it has been observed that prostate cancer presents a high grade and advanced stage when UII-R expression intensity is high (146). Such contradictory findings call for further research to clarify the matter.

### Renal Cancers

In the context of kidney cancer, human renal cell carcinoma markedly proliferates under UII therapy (143), while bladder cancer prognosis is better when UII-R is expressed at heightened levels (145). One study reported that non-muscle invasive and muscle invasive bladder cancer could be differentiated based on UII-R expression in 130 tissue specimens of bladder cancer (145). Furthermore, it has also been proposed that bladder cancer is modulated with UII-R participation, with bladder cancer cells being potentially dependent on UII/UII-R mediated pathway for movement, infiltration, and progression (147).

### Gastrointestinal Cancers

#### 1-Liver Cancer

The heightened expression of UII and UII-R mRNA and protein promotes the growth of human HCC by activating the PKC, ERK1/2, and p38 MAPK signaling pathways (148). Furthermore, UII stimulates the release of reactive oxygen species, which seem to not only accompany the activation of the PI3K/Akt and ERK signaling pathways but also speed up the G1/S transition, which could be the mechanisms underpinning UII-mediated ROS in the promotion of proliferation of cells (149). It is also worth noting that, by encouraging VEGF to be produced and by intensifying HCC tumor growth and progression, UII may participate in tumor angiogenesis (85). It has been observed that HCC tissue has considerably high levels of UII and UII-R mRNA expression (85), which may activate migration and invasion within HCC as well (150, 151). Furthermore, HCC is more likely to recur, invade, and metastasize following radical therapy due to the poor prognosis. It has also been noted that, in HCC cases, UII-R expression is favorably related to tumor number, volume, histology, node metastasis, recurrence, and mortality. Hence, prognosis in HCC cases subjected to radical liver resection may be estimated based on UII-R level (151).

#### 2-Colorectal Cancer

One study evidenced that, by contrast to healthy colon, colon adenocarcinomas had significantly higher expression of UII-R protein and mRNA; in particular, the expression of UII-R mRNA was eight times higher in colon cancer. On the one hand, UII stimulated colon cancer cells to grow, whilst on the other hand, cell growth, motility, and infiltration were suppressed when certain antagonists were used to block UII-R. It can thus be concluded that colon cancer may develop with the involvement of the UII-R-related pathway and treatment benefits could be derived from targeting UII-R (152).

### Lung Cancer

Research has indicated that human lung adenocarcinoma A549 cells have expression of both UII and UII-R mRNAs and proteins. Furthermore, those cells proliferate at a heightened level when UII is administered. One study using nude mouse models of human lung adenocarcinoma A549 cells discovered that UII treatment was associated with a marked increase in the volume and weight of the tumor by contrast to models without UII treatment. Such results support the fact that human lung adenocarcinoma may be promoted by UII in the form of an autocrine/paracrine growth stimulating factor (153).

## Conclusion and Future Perspectives

**Figure 1:**
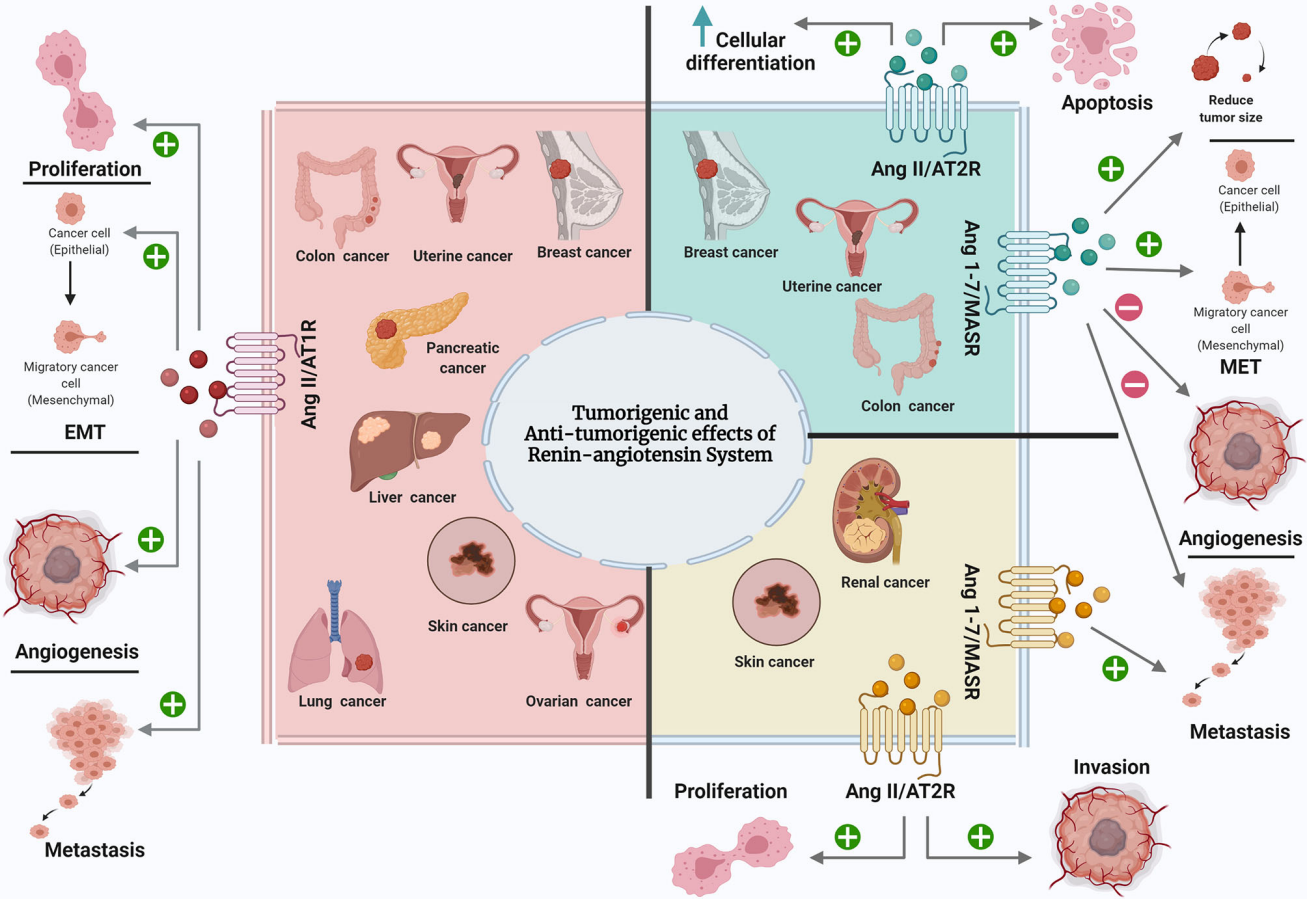
The effect of RAS components on the most common types of cancer. Ang II, Angiotensin II; AT1R, Angiotensin II type 1 receptor; Ang 1-7, Angiotensin 1-7; AT2R, Angiotensin II type 2 receptor; MASR, MAS Receptor; EMT, epithelial mesenchymal transition; MET, mesenchymal epithelial transition. Created with: biorender.com.

In this review, the effect of RAS components on the most common types of cancer was discussed (**Figure 1**). Throughout, it was identified that RAS play a pivotal role in the most common cancers, as it is expressed locally in all normal and cancerous tissues. Dysregulation of RAS components in tumours caused diverse effects on each type of cancer and sometimes even on different stages of the same cancer type. In the majority of studies, Ang II was found to promote cancer progression, growth, EMT, angiogenesis, and metastasis in breast, lung, uterine, prostate, renal, liver, colorectal, pancreatic, and esophageal cancer, chiefly through AT1R and the inhibition of cancer progression, growth, and angiogenesis, inducing cell differentiation and apoptosis in breast, lung, uterine, prostate, renal, liver, ovarian, and pancreatic cancer through AT2R. In contrast, Ang 1-7 inhibits cancer progression, growth, cell motility, angiogenesis, invasion, metastasis, tumour size and weight in breast, lung, prostate, ovarian, and liver cancer. Few studies reported the antitumourigenic effect of Ang II/AT1R, as well as the tumourigenic effect of Ang II/AT2R and Ang 1-7/MASR in cancers. As in case of skin cancer, Ang II supports cancer progression through both Ang II receptors. At the same time, AT1R has dual effects: the aforementioned tumorigenic effect and the Ang II-independent antitumorigenic effect. The dualism of AT1R function during skin cancer may partially explain why the use of ARB in some clinical and preclinical studies of skin cancer promoted cancer growth. Moreover, in the case of kidney cancer, Ang 1-7 – through the MASR – promotes cancer metastasis, which could explain the reason behind the increased risk of kidney cancer among ACEI users, where the ACEIs prevent Ang II production, thus promoting Ang I’s conversion to Ang 1-7 and causing carcinogenesis in kidney cancer. For each case of cancer, and before a decision on whether ARBs and ACEIs are suitable as prophylactic agents, adjuvant therapies, or in combination with other drugs, it is necessary to consider each part of RAS and how it interacts in this type of cancer, or at this stage of cancer.

In conclusion, the ACE/Ang II/AT1R axis has a tumourigenic role, while the ACE-2/Ang 1-7/MASR axis has an antitumorigenic role in most cancers. Moreover, ACEIs and ARBs mostly improve cancer outcomes. In future, more studies concerning the relationship between RAS components and cancer mechanisms are warranted, as are investigations focusing on the role of ACEIs and ARBs in different cancers in large population, with a long follow-up duration, and the adjustment of potential confounding factors. Further studies are also necessary to determine the optimal dose-response, as ACEIs and ARBs exhibited a positive impact in most studies, increasing the likelihood of them accelerating the development of cancer therapy.

Additionally, knowledge is limited about how UII contributes to tumor biology. UII participation in the pathophysiology of a wide range of conditions determines the perspectives for the development of UII-R inhibitors to treat various cancers (e.g. prostate, liver, colon cancer) in which heightened UII/UII-R activity encourages tumor cells to growth, migrate, and infiltrate. Moreover, future research stands to gain from both basic experimental studies on UII and UII-R and large-scale clinical studies.

Research is also needed to establish the levels of expression of UII and its receptor in certain cancers (e.g. ovarian, pancreatic, esophagus, skin cancer). Such research can corroborate the findings from earlier work that identified UTR as a new cancer prognosis biomarker. In addition, it has been observed that, in the context of angiogenesis, UII and Ang-II exhibit synergistic action in promoting VEGF production in adventitial fibroblasts, which is a major step in tumor angiogenesis (141). However, the synergy of UII and Ang-II has not been investigated in relation to cancer. Therefore, new targets for treatment could be discovered by examining the possible mutual effect of UII and Ang-II or the nature of their interaction in the context of cancer progression.

## Author Contributions

MA wrote the manuscript. TB design and reviewed the manuscript. AbA and AmA edited and reviewed the manuscript. HA reviewed the manuscript. All authors contributed to the article and approved the submitted version.

## Funding

This review article was supported by grant from King Abdullah International Research Center no. RC18/171/R.

## Conflict of Interest

The authors declare that the research was conducted in the absence of any commercial or financial relationships that could be construed as a potential conflict of interest.

## Publisher’s Note

All claims expressed in this article are solely those of the authors and do not necessarily represent those of their affiliated organizations, or those of the publisher, the editors and the reviewers. Any product that may be evaluated in this article, or claim that may be made by its manufacturer, is not guaranteed or endorsed by the publisher.

## Glossary

**Table:**

RAS	renin angiotensin system
AGT	angiotensinogen
Ang I	angiotensin I
ACE	angiotensin converting enzyme
Ang II	angiotensin II
AT1R	angiotensin II type 1 receptor
AT2R	angiotensin II type 2 receptor
Ang 1-7	angiotensin 1-7
MASR	MAS Receptor
ACE-2	angiotensin converting enzyme 2
VEGF	vascular endothelial growth factor
EMT	epithelial mesenchymal transition
MMPs	matrix metalloproteinases
ECM	extracellular matrix
ACEIs	ACE inhibitors
ARBs	AT1R blockers
PPAR-γ	peroxisome proliferator-activated receptor-gamma’s
CBM	CARMA3-Bcl10-MALT
FAK	focal adhesion kinase
RhoA	Ras homolog gene family member A
IL-6	interleukin-6
ATIPs	AT2R interacting proteins
TAM	Tamoxifen
ER	estrogen receptor
GLPM	glycolipid-based polymeric micelles
GLPM/Tel	Telmisartan loaded GLPM
CAFs	cancer-associated fibroblasts
MCS	multicellular spheroids
SREBP	sterol regulatory element-binding protein
SCD1	stearoyl-CoA desaturase-1
HMGA2	high-mobility group, AT-hook 2
EC	endometrial cancer
PC	prostate cancer
PTK	protein tyrosine kinase
RCC	renal cell carcinoma
HCC	hepatocellular carcinoma
ROS	reactive oxygen species
AMPK	AMP-activated protein kinase
mTOR	mammalian target of rapamycin
CRC	colorectal cancer
KCs	Kupffer cells
PFS	progression-free survival
AP-1	activating protein-1
PDAC	pancreatic ductal adenocarcinoma
miR-410	microRNA 410
ESCC	esophageal squamous cell carcinoma
EAC	esophageal adenocarcinoma
CSC	cancer stem cells
NSCLC	non-small cell lung cancer
EGFR	epidermal growth factor receptor
miRNA-21	micro RNA-21
MAPK/STAT	mitogen-activated protein kinase/signal transducer and activator of transcription
TKIs	tyrosine kinase inhibitors
TRAIL	tumour necrosis factor-related apoptosis-inducing ligand
LSCC	lung squamous cell carcinoma
MDSC	myeloid derived suppressor Cells
NEH1	N^+^/H^+^ Exchanger Isoform1
HNSCC	head and neck squamous cell carcinoma
